# Older Peoples’ Adherence and Awareness of Changes in Drug Therapy after Discharge from Hospital

**DOI:** 10.3390/pharmacy6020038

**Published:** 2018-05-02

**Authors:** Sara Norberg, Maria Gustafsson

**Affiliations:** Department of Pharmacology and Clinical Neuroscience, Umeå University, SE-90187 Umeå, Sweden; sara.norberg@vll.se

**Keywords:** adherence to medications, older people

## Abstract

Non-adherence is important to address because it might affect the effectiveness of therapy and lead to adverse effects. The objectives of this interview study were to investigate old peoples’ general adherence to drugs and their awareness of and adherence to changes in drug therapy after their hospital stay. Following ethical approval, 42 patients admitted to the medical ward were invited to participate in this study. Of these, 36 persons, with a mean age of 82.5 years, who were discharged to their home, were interviewed by telephone using the Medical Adherence Report Scale (MARS) to assess their general adherence to prescribed drugs. Questions regarding awareness and adherence to drug changes during their hospital stay were asked. Different factors related to adherence and non-adherence were investigated using the Pearson chi-square test and the independent sample t-test. The average MARS score was 23.9 ± 1.4, with 31 persons (86%) assessed as adherent to their drug therapy and 5 persons (14%) as non-adherent. Of the 36 people, 30 had at least one change in their drug therapy during their hospital stay, and 23 (77%) of these people were aware of all changes and 23 (77%) were adherent to all of the changes. No significant differences between adherence and age, gender, living situation, or number of drugs were found. This small study found that some older people who were discharged from hospital were generally non-adherent, and some were not aware of or adherent to changes made in the drug therapy during their hospital stay. This is an important problem to address with further interventions.

## 1. Introduction

Life expectancy in Sweden is one of the longest in the world. A contributing factor to increased life expectancy and improved health is drug treatment. On average, half of all persons over 75 years in Sweden are prescribed 5–6 different drugs, and about 15% of those who are 80 years of age or older have 10 or more drugs. The risk of unwanted effects of drug treatment increases with increasing age, and an increased number of drugs is also considered to be associated with reduced adherence to drug treatment [1].

According to the World Health Organisation (WHO), the definition of adherence is: “the extent to which a person’s behaviour—taking medication, following a diet, and/or executing lifestyle changes, corresponds with agreed recommendations from a health care provider” [2]. That the person takes the correct drug at the right dose at the correct intervals is an important factor associated with the effectiveness of the drug treatment [3]. A systematic review concluded that adherence to medications is complex and that many factors such as age, poor follow-up, and multiple drugs are associated with non-adherence [4]. Not being adherent to drug treatment can also be an active decision from the person based on personal beliefs about possible risks and benefits of drug treatment [5]. If non-adherence in a patient is not observed, this might result in a dosage increase or in additional medication being prescribed, which in turn might lead to increased risk of unwanted effects and even readmissions to hospital [6]. An indirect method that is relatively easy to use to measure drug adherence is self-reporting through surveys or patient interviews [7]. An example of such a method is the Medication Adherence Report Scale (MARS), a validated Likert-type scale [8].

Low adherence might have a major effect on treatment outcomes, contribute to disease impairment, increase healthcare costs, and even lead to death [7]. Numerous studies regarding adherence to drugs have been conducted, for example, one Swedish study that found non-adherence among 35% of people using antihypertensive therapy [9]. However, adherence and awareness of changes in drug therapy after discharge from hospital in Sweden have received limited attention in medical literature. The aim of this study was therefore to investigate the general drug adherence and associated factors among older people living at home after being discharged from a medical ward at Umeå University Hospital in Sweden. A second aim was to assess awareness and adherence to drug changes made in the patients’ drug therapy during their hospital stay, after being discharged from the hospital. 

## 2. Materials and Methods 

### 2.1. Material and Procedures

This interview study included people aged ≥70 years who had been discharged from the medical ward at Umeå University Hospital in Sweden. A clinical pharmacist met the patients the same day as they were discharged and invited them to participate. People who were invited to participate were aged ≥70 years and discharged from the ward to ordinary housing with at least one regular medicine prescribed. A clinical pharmacist conducted the interviews by telephone within a week after discharge. Those excluded were palliative patients, patients who could not speak for themselves, patients with no personal responsibility for their medications, and patients admitted to hospital due to alcohol or drug intoxication.

The medical histories of the study participants were retrieved from their medical records. Patient background included age, gender, and information regarding medical treatment. Data about the drug changes made during hospitalisation was also obtained from the medical records. In the interviews, the MARS was used to assess the general adherence and questions about adherence and awareness of drug changes during hospitalisation were asked. The patients were asked if they knew if any changes in drug therapy had been done during their hospital stay (yes or no). If changes in drug therapy had been done, each change was discussed with the patient to assess whether the patient was adherent and/or aware of the change. Additionally, questions were asked about drug management, and practical problems with the medications or with the packaging. 

### 2.2. Definitions

MARS scores, ranging from 5–25, were used to assess the general adherence [8]. Patients with a score ≥23 were defined as adherent, while people with a score ≤22 were defined as non-adherent [5]. A person was required to be aware and adherent to all changes in drug therapy during their hospital stay to be defined as aware and adherent of these. 

### 2.3. Data Analysis

Different factors related to adherence and non-adherence were investigated using the Pearson chi-square test and the independent sample *t*-test. Awareness of drug changes during hospitalisation among patients who did and did not receive a discharge message was compared using the Pearson chi-square test. A *p*-value of <0.05 was considered statistically significant. Statistical calculations were performed using the SPSS v.23 software.

### 2.4. Ethical Approval and Trial Registration

The regional Ethics Review Board in Umeå approved the study (nr 2016/452-31). People participating in the study were given written and orally presented information about the research. Informed consent was obtained from all individual participants included in the study before the interviews were conducted.

## 3. Results

Between 1 February 2017 and 31 March 2017, 42 patients admitted to the medical ward were invited to participate in this study. Three patients declined participation. The remaining 39 patients were included before discharge. Of these 39 patients, one used the right to withdraw after discharge, one did not answer the telephone despite several attempts, and one person was readmitted to hospital before the interview. The data from the remaining 36 persons was included in the analysis.

The average age of the study participants was 82.5 years, 56% were women, and the mean number of regularly used drugs was 7.4. Of the 36 persons included, 9 (25%) had help with their drugs either from a close relative or from municipal healthcare services (Table 1). 

The mean MARS score was 23.9 ± 1.4 (range, 19–25), and 31 persons (86%) were considered to be adherent (MARS ≥ 23) while 5 persons (14%) were considered non-adherent (MARS ≤ 22). No significant differences between adherence and age, gender, living situation, or number of drugs were found (Table 2).

Of the 36 participants, 30 (83%) had at least one drug change during their hospitalisation. The number of drug changes during the hospitalisation was on average 3 ± 2 (range, 1–11). Of the 36 persons, 23 (77%) were aware of all drug changes and 23 persons (77%) were adherent to all drug changes. Of the seven persons (7/30, 23%) judged to be non-adherent, two were aware and five were not aware of the drug changes. Of the two persons who were aware of the changes but were not adherent, one person (20%) did not pick up their prescription from the pharmacy and one person (20%) used a too-low dose of the drug. Among the five persons not aware of the changes, four (80%) had misunderstood the changes and one (20%) did not participate in the treatment.

Of the 23 persons who were aware of all drug changes during their hospitalisation, 18 (18/23, 78%) had received a written discharge message from the ward that described all drug changes. Among the seven persons not aware of the changes, five (5/7, 71%) had received a written discharge message. This study could not demonstrate that this message affected the patients’ awareness of drug changes during their hospitalisation (*p* = 0.708).

## 4. Discussion

This is a small pilot study, which is why the results can only give an indication of patients’ general level of adherence to medications, and awareness and adherence to drug changes during their hospital stay. This study found that according to MARS, 14% of the persons were non-adherent to their drug treatment. This is in line with a previous study using MARS in the form of a questionnaire [5]. Previous studies have shown that there are several factors that might affect patient adherence, for example, old age, female sex, civil status, and multiple drugs [4]. Such associations were not found in this study, perhaps due to the small number of patients. 

This study found that drug changes during hospitalisation were common, and this was also found in previous research [10]. The present study found that 77% of the patients were adherent to all drug changes made during hospitalisation, and this is slightly better than what was found in a study performed in Italy, where the adherence of older patients with polypharmacy who were recently discharged from hospital was less than 50% [11]. Nevertheless, this is an important finding that needs to be emphasised. 

Most of the patients who were aware of the drug changes were also adherent to these changes. It seems that those who might be at the greatest risk of further problems are those who are not aware of the changes and for that reason are not adherent to the changes. It is important to detect and pay attention to these individuals and to ensure that they receive comprehensive information about the drug changes that are made. One way is to use a written discharge note, which a high percentage of the study participants had received. Even if no significant difference regarding awareness was found between those who did or did not receive such a note, all people discharged with drug changes should probably get a discharge letter. In addition, one way to improve the information from the physicians might be to provide information to the patient from clinical pharmacists before or after discharge. If a clinical pharmacist calls the patients after discharge and discuss the changes in medications, this might increase adherence and awareness of changes done. More research is needed to investigate this further. 

A limitation of this study is the small number of study participants. As a consequence, the representativeness of the study population is low. There might be risk of bias and chance findings and the chance to find statistically significant relationships is very low due to the limited number of observations. This is also the reason why only a few analyses were conducted in this pilot study. In a larger study, additional analyses such as associations between non-adherence and the number of changes made to the existing regimen or number of medicines would be interesting to investigate. In conclusion, for these reasons, the results in this study should be interpreted with caution. Further, people who were not able to speak for themselves or with no personal responsibility for their medication were excluded. This excluded many people with cognitive impairment, a group of people who might have reduced adherence to drugs [7], meaning that the problems with adherence are probably underestimated in this study. One of the strengths with this study is that the chosen assessment of general adherence, MARS, is a validated scale [8]. Using self-reporting as a method is simple, inexpensive, and useful in clinical practice, but it tends to overestimate patient compliance [7]. Combining different methods increases the precision of the measurement [7], and this is why follow-up studies should include both self-reporting and register studies.

## 5. Conclusions

This small study found that some older people who were discharged from hospital were generally non-adherent, and some were not aware of or adherent to changes made in the drug therapy during their hospital stay. This is an important problem to address with further interventions. 

## Figures and Tables

**Table 1:** Characteristics of participants.

Number	36
Gender (%)	
Women	20 (55.6)
Men	16 (44.4)
Age, mean ± SD (range)	82.5 ± 6.6 (70–93)
Number of drugs taken regularly, mean ± SD (range) Living arrangement (%)	7.4 ± 3.1 (2–13)
Living alone	14 (38.9)
Living with a relative	22 (61.1)
Drug management (%)	
Help with medication*	9 (25.0)
Pill organizer	28 (77.8)
Dose-dispensed drugs	1 (2.8)
Practical problems with the medication **	19 (52.8)
Problems with the packaging	13 (36.1)

* From relatives or from health care ** For example, to split the tablet into two parts.

**Table 2:** Adherence according to Medical Adherence Report Scale (MARS).

	Adherent (MARS ≥23) n = 31	Non-Adherent (MARS ≤22) n = 5	p-Value
Age, mean ± SD	82.3 ± 6.4	83.2 ± 8.0	0.794
Gender (%)			0.451
Women	18 (90.0)	2 (10.0)	
Men	13 (81.2)	3 (11.5)	
Living arrangement (%)			0.297
Living alone	11 (78.6)	3 (21.4)	
Living with a relative	20 (90.9)	2 (9.1)	
Number of drugs taken regularly, mean ± SD	7.3 ± 3.1	8.2 ± 3.7	0.554
